# Protein Tau: Prime Cause of Synaptic and Neuronal Degeneration in Alzheimer's Disease

**DOI:** 10.1155/2012/251426

**Published:** 2012-06-08

**Authors:** Natalia Crespo-Biel, Clara Theunis, Fred Van Leuven

**Affiliations:** Experimental Genetics Group (LEGTEGG), Department of Human Genetics, KU Leuven, Campus Gasthuisberg ON1-06.602, Herestraat 49, 3000 Leuven, Belgium

## Abstract

The microtubule-associated protein Tau (MAPT) is a major component of the pathogenesis of a wide variety of brain-damaging disorders, known as tauopathies. These include Alzheimer's disease (AD), denoted as secondary tauopathy because of the obligatory combination with amyloid pathology. In all tauopathies, protein Tau becomes aberrantly phosphorylated, adopts abnormal conformations, and aggregates into fibrils that eventually accumulate as threads in neuropil and as tangles in soma. The argyrophilic neurofibrillary threads and tangles, together denoted as NFT, provide the postmortem pathological diagnosis for all tauopathies. In AD, neurofibrillary threads and tangles (NFTs) are codiagnostic with amyloid depositions but their separated and combined contributions to clinical symptoms remain elusive. Importantly, NFTs are now considered a late event and not directly responsible for early synaptic dysfunctions. Conversely, the biochemical and pathological timeline is not exactly known in human tauopathy, but experimental models point to smaller Tau-aggregates, termed oligomers or multimers, as synaptotoxic in early stages. The challenge is to molecularly define these Tau-isoforms that cause early cognitive and synaptic impairments. Here, we discuss relevant studies and data obtained in our mono- and bigenic validated preclinical models, with the perspective of Tau as a therapeutic target.

## 1. Introduction

It is no coincidence to begin this paper on protein Tau and tauopathies by referring to Alzheimer's disease (AD), because it is the most frequent tauopathy, albeit a “secondary” one. More than hundred years after the first documented case of AD, the etiology of sporadic forms remains hitherto unknown. Demographic changes, increased life expectancy with a fast growing elderly population, lead us and all future generations to the dramatic increase in the incidence and prevalence of AD—described by some as “the pandemic of the 21st century” [1].

The extracellular amyloid deposits, c.q. neuritic plaques and cerebrovascular angiopathy, together with the intracellular neurofibrillary threads and tangles (NFT) remain the key pathological markers in AD brain. Nevertheless, the essential basis of cognitive impairment is generally accepted to be reduced synaptic plasticity, evolving into loss of synapses and eventually loss of neurons only in later stages [2–5]. Extensive genetic and biochemical data have implicated the amyloid peptides, the main components of the amyloid deposits, as the central mediators of AD. The amyloid cascade hypothesis, prevailing for more than two decades, stated that the amyloid deposition is the initiating event of neuronal dysfunction and cell death in brain [6]. In familial AD-cases, production of more and longer amyloid peptides, for example, A*β*1–42, by abnormal proteolysis of the membrane-bound amyloid precursor protein (APP) triggers a pathologic cascade, years, or decades before the overt clinical manifestations of the disorder [7–9]. By extension, a similar pathogenic pattern is proposed in sporadic AD cases, whereby increased levels of similar or other amyloid peptides, for example, A*β*1–38, A*β*1–43, pE3A*β*3-x among others, can stem from deviating proteolysis, increased production, failing degradation, and ApoE-mediated elimination from the brain.

The major—if not only—arguments that support the amyloid hypothesis are genetic by nature [12]. The detection of different mutations in the gene coding for APP, but even more so in the gene coding for presenilin-1 (PS1) is defined as causing early onset familial AD [12]. Conversely, overproduction of amyloid peptides from mutant APP, alone or in combination with mutant PS1 in transgenic mouse brain, failed to cause appreciable tauopathy or neuronal degeneration. The question arose whether accumulating amyloid peptides are indeed the real culprit for neurodegeneration in AD. Recent neuroimaging studies demonstrated considerable amyloid deposits in the brain of a sizeable fraction, that is, 15–30% depending on the study, of cognitively normal individuals, whereas clinically diagnosed AD patients can show no amyloid deposition by PET scan [13, 14]. These and other observations have led to the modified amyloid hypothesis, stating that not deposits but soluble toxic amyloid species must initiate AD-pathology, and moreover, that tauopathy is essentially implicated [15].

For decades, the microtubule-associated protein Tau was known as the main component of NFT, and although occupying the number two spot on the list of proteins implicated in AD, the focus of the field is traditionally aimed on amyloid. Long-standing observations, nevertheless, indicate that neuronal tauopathy provides the closest pathological approximation of clinical defects observed during the life of AD patients. The typical brain-regional occurrence and progression of Tau pathology correlates temporally and spatially well with neuronal and cognitive dysfunction [16, 17], with the added weight of the correlation to CSF-levels of phosphorylated Tau [18]. Therefore, the crucial question remains where protein Tau should be placed in the amyloid cascade hypothesis: downstream, at the same level or even upstream of amyloid? Should we consider protein Tau as a by-stander of the amyloid toxicity, as a mediator or even as a prime player, and therefore preferred target? Only recently fundamental scientist have gained more insight into how these two main defects and players could be linked mechanistically.

Important is that even in young familial AD-cases, amyloid pathology is always accompanied by Tau pathology, similar to late sporadic cases of AD. This establishes tauopathy even more firmly as cause, besides or rather than codiagnostic hallmark in all AD cases! Scientific reasoning takes this one step further, to implicate tauopathy in the more early stages of AD, conform to the origin of pathology in primary tauopathies. Important caveat here is that the early stages of Tau pathology are yet to be defined molecularly in human patients. This was and remains a formidable scientific challenge: biochemical and biophysical definition of the neurotoxic Tau species, which we have termed Tau-P* [19].

## 2. Protein Tau: Physiology and Pathology, an Unstable Balance

In contrast to the apparent redundancy of the physiological function of protein Tau, as demonstrated in Tau-deficient mice [20], the presumed pathological importance of protein Tau was established by the discovery of inherited mutations in the MAPT gene. These are tightly linked to the disease in families suffering autosomal dominant frontotemporal dementia with parkinsonism linked to chromosome 17 (FTDP-17) [21–24]. Once more, genetic findings established a key factor in the pathogenesis of a disease, in this case protein Tau in a subgroup of FTD, a heterogeneous group of tauopathies characterized by dementia and movement disorders.

The structure of the Tau gene has been reviewed in detail elsewhere [25, 26]. Alternative mRNA splicing of exon 10, encoding the second of four microtubule-binding domains (MTBD) produces two major isoforms, denoted as Tau.3R and Tau.4R, respectively, with lower and higher affinity for microtubules (MT) [27–29]. The abundance of Tau.3R and Tau.4R in human brain changes during brain development and neuronal differentiation. In embryonic brain, Tau.3R isoforms dominate, apparently providing structural and morphological plasticity to developing and differentiating neurons. In mature neurons, Tau.4R gains importance and becomes largely located in axons, excluded from soma and dendrites where other MAPs predominate. In the adult human brain, Tau.3R and Tau.4R isoforms are balanced, while in adult mouse brain the Tau.4R isoforms prevail [30, 31].

Importantly, both intronic and exonic mutations in the MAPT gene are associated with FTDP-17. Many are located in exon 10 or in the surrounding introns, affecting the alternative splicing of this exon. Evidently, intronic mutations produce normal, wild-type proteins, which in case of protein Tau result in the distortion of the normal isoform-balance. An abnormal Tau.3R/4R balance, either increased or decreased, is thought to disturb the function of Tau in MT assembly, stabilization, and transport. Expressed mutations then are presumed to provoke similar problems, while mutant Tau in general is more prone to phosphorylation and polymerization [27–29].

Immunohistochemical and biochemical studies, supported by genetic data, have revealed that in specified tauopathies the pathology directly reflects the aberrant Tau isoform ratio. For instance, in PSP, CBD, and AGD, the inclusions contain mainly Tau.4R isoforms [30, 32, 33], whereas Pick bodies contain mostly Tau.3R. Furthermore, in familial FTD, exonic mutations, for example, P301L, P301S, G272V, N279K, V337M, R406, cause significant overrepresentation of Tau4R [34, 35]. In AD and other tauopathies, variable ad-mixtures of the two major Tau isoforms are biochemically detected in aggregates and deposits in soma, dendrites, and axons. Obviously, an inconsistent picture emerges of Tau isoforms biochemically associated with different tauopathies, although all are pathologically defined by argyrophilic tauopathy.

Our eventual molecular understanding of the role played by abnormal structural features of protein Tau in human tauopathies must ultimately be based on the knowledge of its normal cellular functions. The major, and as far as we know, only physiological function of protein Tau involves its capacity to bind to microtubules to control or affect spacing, stabilization and dynamics [36–38]. That protein Tau is involved in signal transduction, neuronal differentiation, organello-genesis and growth can likely be reduced to the same primary act of MT-binding [39–41]. We will not indulge in describing the processes in which protein Tau has been implicated or proposed, a subject reviewed recently elsewhere [42].

The function of Tau in adult—and ageing—brain is supposed to be fine-tuned by post-translational mechanisms besides the alternative mRNA splicing producing Tau.3R and Tau.4R isoforms. The prime one is phosphorylation of a limited set of serine and threonine residues, selected among the 79 available. With respect to the mechanism governing this selection, one must not only consider the specificity of different kinases responsible, but also the naturally unfolded nature of protein tau, which presumably exposes most, if not all the S/T residues.

## 3. Phosphorylation, Missorting, and Aggregation: In What Order and Extent?

Over the last years, following and thanks to the genetic discoveries in the MAPT gene, striking observations in patients but mainly in experimental models have changed some long-established convictions concerning the role of protein Tau in cognitive disorders and neurodegeneration.

In transgenic mice, regulated expression and suppression of mutant protein Tau correlated with defective and improved cognition [43]. Moreover, protein Tau but not NFT appeared involved in neuronal death in transgenic mice and AD patients [44, 45]. Elimination of protein Tau in an amyloid mouse model restored cognitive and behavioral deficits [46], corroborating observations in AD patients that tauopathy defines the cognitive demise, discussed above. Moreover, not APP but protein Tau proved neurotoxic for pyramidal neurons in CA1 and cortex in a nontransgenic, AAV-based model, with formation of oligomeric but not fibrillar Tau [47].

 Ideas that have been simmering for years are more and more substantiating that specified neuronal dysfunctions are evident before NFTs are deposited. Moreover, the synapto- and neurotoxicity of protein Tau depends on its posttranslational modifications, in first instance O-phosphorylation, but also others that need to be explored in detail: Y-phosphorylation, glycation, glycosylation, acetylation, sumoylation, among others [48–52]. Therefore, accurate temporal and spatial patterns of pathological traits that must also be physiologically important deserve investigation to understand their role in the genesis and evolution of AD and tauopathies.

In general, increased phosphorylation of protein Tau decreases its binding to MT and thereby regulates the normal biological activity of protein Tau related to microtubule spacing, assembly and stability. Obviously, phosphorylation of protein Tau is part of normal physiological regulatory processes and not pathogenic per se [39, 53]. Conversely, in AD and all primary tauopathies, protein Tau becomes more and more phosphorylated with disease progression, to enter a state that is generally referred to as “hyper-phosphorylated.” Nevertheless, no exact lower or upper limits are defined to this connotation, which exposes a major molecular and analytical problem: how many of the vast number of potential O-phosphorylation sites in protein Tau are physiologically relevant? In second order, the question is, are these sites directly pathologically implicated—or merely correlated? We currently address these questions by “timeline” analysis of transgenic mouse brain to define which phosphorylation levels and sites generate or accelerate disease.

While several studies suggest that the functional impact of phosphorylation depends on the specific phosphorylation sites [54–56], others support the notion that overall increased, not site-specific, phosphorylation is sufficient [57]. It is well-known that in development and postnatally, protein Tau is heavily phosphorylated. This “fetal Tau” binds less to MT and helps maintain dynamics of microtubule assembly/disassembly during this period of most active neurite outgrowth, albeit without eliciting tauopathy or neurotoxicity as in ageing brain. Differentiating functional differences between normal Tau in the immature brain and pathological Tau in ageing brain might define phosphorylation of Tau at some specific sites, for example, S199, T231, and S396 in AD brain [58].

Tau phosphorylation is markedly increased in response to various stressors. Phosphorylation, although to lesser levels than in AD brain, appears to be mobilized by neurons to regulate activity of Tau transiently and reversibly as required. A prime example is hibernation as adaptive process that represents a powerful physiological strategy to withstand periods of limited energy supply. Hibernation is a hypometabolic state with declining body temperature, periodically interrupted by brief spontaneous periods of rewarming to core temperatures, whereby selective expression and control of kinases and phosphatases is an adaptive response for long-term survival [59]. Highly phosphorylated protein Tau readily accumulates and even appears to aggregate in brain of hibernating animals, while most interestingly the entire process is fully reversed when animals arouse, restoring normal temperature and metabolism without harming neurons or networks [60–62].

Other conditions, associated with reduced body temperature and increased Tau phosphorylation are starvation [63], cold water stress [64], and anesthesia [65]. Several reports suggest that anesthesia-induced hypothermia increases the risk of AD [66, 67]. Temperature fluctuations affect the relative balance of kinase and phosphatase activity [65, 68]. In a mouse model with incipient neurofibrillary pathology, the pool of free hyperphosphorylated Tau was recruited into Tau aggregates that accumulate over time [69]. Moreover, isoflurane anesthesia in young Tau.P301L transgenic mice provokes brainstem tauopathy and upper airway defects, suggesting similar problems in elderly patients exposed to anesthetics for surgery [70].

## 4. Tau Kinases: GSK3 Accepted, Others Explored…

The GSK3 kinases appeal as candidates to modulate Tau phosphorylation for several reasons: (i) they are abundantly expressed in neurons, (ii) their levels and/or enzymatic activity is increased in AD brain [71, 72], and (iii) they can phosphorylate many of the S/T-P sites of protein Tau that are also phosphorylated in AD brain, that is, S199, T231, and S396 [73–75]. Additionally, in our transgenic Tau mouse models, GSK3 greatly increased the severity of tauopathy when expressed in conjunction with mutant Tau.P301L [76, 77], but alleviated the axonopathy when coexpressed with wild-type Tau.4R [78]. Moreover, and surprising for a naturally unfolded protein is the notion that phosphorylation affects the conformation of protein Tau. Monomeric Tau in solution, when phosphorylated at critical sites either upstream or downstream of the MTBD, appeared to adopt a more compact conformation that was proposed to reflect its propensity to aggregate [79].

The long-standing definition of protein Tau as an axonal protein, and even axonal marker [80, 81], contrasts with the early pathological defects observed in most tauopathies: abnormal delocalization into the somatodendritic compartment where protein Tau becomes even more phosphorylated and subsequently forms the well-known aggregates and NFT. Several hypotheses for the axon-specific location were proposed: axon-specific sorting of mRNA coding for protein Tau or preferential degradation of mRNA and/or protein Tau in dendrites, affected or even steered by selective phosphorylation of protein Tau [82–84]. In the last years, among efforts to clarify these issues a new proposal for a retrograde filtering action dependent on microtubules would allow protein Tau to move into but not out of axons [85]. Moreover, once protein Tau becomes phosphorylated, its microtubular interaction is decreased or disrupted, and phospho-Tau can bypass that barrier. A different mechanism depends on the activation of the PAR-1/MARK kinase to phosphorylate protein Tau downstream of signaling initiated by amyloid [86], thereby contributing to relocate protein Tau. This would explain its effects on synaptic trafficking, anchoring of glutamate receptors, and interaction with other kinases in disturbing postsynaptic functions [40, 87].

## 5. The Neurotoxic Species, Known Only as Tau-P*

Intracellular aggregation and deposition of protein Tau as NFT in soma and processes is commonly observed in all tauopathies, including AD. These aggregates, predominantly composed of highly phosphorylated protein Tau, are detergent insoluble and presented either as paired helical filaments (PHF), twisted ribbons, or straight filaments, usually in varying combinations. Although the load of NFT correlates with the severity of cognitive impairment in humans, mouse models for tauopathy demonstrate memory defects to precede NFT-like tauopathy [19, 77, 88, 89]. Moreover, in a conditional model, reversal of Tau expression reversed the cognitive defects even without affecting the Tau deposits [43, 90]. The levels of early multimeric Tau-aggregates that preceded the NFT were found to correlate with memory deficits [91]. These findings corroborate the hypothesis that molecularly unspecified intermediates in the Tau-aggregation cascade, situated between single Tau molecules and large fibrils, are the actual neurotoxic agents. The connotation Tau-P*, as defined previously [19, 92], represents intermediate forms of phosphorylated protein Tau, which remain to be defined molecularly. They are proposed to adopt a transitional conformation state and to be the effective executers of synaptic and eventually neuronal toxicity, if or when they are not directed into formation of the large fibrillar Tau-aggregates that we now consider not or less harmful for neurons. Therefore, decoding the sequence of events that transform soluble or MT-bound Tau into a toxic molecular intermediate that only later becomes an inert aggregate is the key to understand ontogenesis and evolution of any tauopathy. In addition, these studies entertain the idea that therapeutic intervention is possible by acting on the expression, phosphorylation, and aggregation of protein Tau.

The formation of NFT involves sequential steps as studied best with recombinant proteins *in vitro* and, because cellular models are still lacking, are corroborated by the timeline of events in transgenic mouse brain *in vivo* [93]. The first step is believed to be the neutralization of the protein Tau-microtubule interaction by phosphorylation by different kinases, increasing the cytoplasmic levels of unbound protein Tau. Moreover, while protein Tau lacks a well-defined structure, the initial phosphorylation promotes an unspecified molecular state that appears favorable for dimerization and aggregation [79, 94]. The adopted presumed *β*-sheet structure involves the MTBD of protein Tau, whereby eventual disulfide bridging, although not generally accepted, could help shift the equilibrium from soluble monomers to higher-order multimers [95, 96]. Subsequently, further association of *β*-fibrillar Tau-multimers leads to filaments that deposit as neuropil threads and tangles in the soma.

Biochemical indices for the occurrence of oligomers were interpreted to represent off-pathway aggregates that do not form filaments [97]. Whether or not hyperphosphorylation of protein Tau is an indispensable event for the initial dimerization remains elusive and debated. *In vitro* studies demonstrated that fibrillar assemblies of protein Tau are not per se phosphorylation dependent [74] and even that the propensity of protein Tau to form multimers is reduced by phosphorylation particularly of S262 by MARK2/Par1 [98]. Moreover, even the inverse order of events was demonstrated in a mouse model for aggregation of protein Tau, because the incipient aggregation was the actual trigger for subsequent phosphorylation of protein Tau [99]. Consequently, defining the molecular identity of the intermediate Tau species referred to as Tau-P*, a hyperphosphorylated, conformational small aggregate, will be a major step forward on the arduous route to the most appropriate therapeutic target in AD, and in primary tauopathies.

## 6. Brain-Regional Propagation of Tauopathy

Anatomical patterns of the pathology in human brain suggest, but do not prove, a progressive spreading of tauopathy with progressing cognitive decline [100]. Initial depositions of phosphorylated protein Tau are first observed in the locus coeruleus, one of several subcortical nonthalamic nuclei that has diffuse projection to the cortex. Subsequently, immunochemical defined phosphorylated protein Tau is observed in entorhinal cortex and in its efferent hippocampal and neocortical regions [101].

This apparent spreading of the tauopathy throughout human brain in a specified regional pattern has been the subject of various hypotheses. Initial evidence for cell-to-cell transfer of protein Tau aggregates came from two studies carried out in cells and *in vivo*. The first demonstrated that fibrils of protein Tau added to the culture medium were taken up by neuronal cells and caused formation of Tau-aggregates in the cytoplasm [102]. The second reported that in mouse models of tauopathy, intracerebral injection of brain tissue extracts containing Tau-aggregates initiated spreading of intracellular Tau-aggregation from the injection site to other brain areas, although not producing neurodegeneration [103]. These experimental studies still remain controversial and need to be confirmed, eventually to define the route and mechanism whereby cytoplasmic aggregates of protein Tau can become secreted and taken up by neurons, in order to establish the transmittance between neurons as a potential mechanism of spreading of tauopathy in human brain [104]. Most recent data from novel mouse models that express Tau.P301L specifically and only in the entorhinal cortex (ERC) corroborate the hypothesis of cell-to-cell spreading of tauopathy, but leaves the responsible mechanisms open for speculation [105, 106].

## 7. Mouse Models for Tau Pathology

To understand the impact of Tau phosphorylation and aggregation in synaptic and neuronal degeneration, fully characterized mouse models that exhibit the typical phenotypic features and pathological changes of human tauopathies are needed. Many transgenic mouse models for tauopathy have been developed and characterized over the last decade and were the subject of some recent reviews [19, 88, 107]. Here, we limit the discussion to our mouse models that represent or recapitulate interesting aspects of the pathology of protein Tau in Tau pathologies, including AD.

### 7.1. Tau.4R Mice

They express full length, wild-type human Tau under control of the mouse Thy-1 gene promoter in the FVB/N genetic background. The advantage of the mouse Thy-1 gene promoter permits neuron-specific expression of the transgene, which begins in the second week postnatally, avoiding interference with development. Tau.4R mice present increased phosphorylation of human protein Tau, however without formation of any Tau-aggregates even at late age [108]. Conversely, Tau.4R mice develop axonopathy, initially consisting first of axonal dilatations or spheroids, evolving into Wallerian degeneration with muscle wasting. The evident motor problems, already present at young age (6 weeks) in homozygous Tau.4R mice, are explained by excessive binding of protein Tau.4R to microtubules. This prevents the normal binding and passage of motor proteins responsible for axonal transport in both directions, resulting in the stochastic accumulation of transported items, from synaptic vesicles to mitochondria over Tau and neurofilament aggregates [108].

Remarkably, the severe axonopathy and motor problems hardly affect the survival of the Tau.4R mice [89, 108]. Moreover, the pathological phenotype was rescued by coexpression of GSK3*β*, resulting in bigenic mice that appear largely normal [78]. Molecularly, this is explained by the evident increased phosphorylation by GSK3*β* of human protein Tau.4R, which displaces it from the microtubules and thereby restores normal axonal transport in both directions. Despite the fact that protein Tau.4R became highly phosphorylated in brain of the Tau.4RxGSK3*β* bigenic mice, no Tau pathology resulted. Besides demonstrating for the first time *in vivo* that GSK3*β* is an effective Tau-kinase, the data also underlined that additional phosphorylation by other kinases is needed to develop tauopathy. A final argument explaining the lack of tauopathy in Tau.4R mice, and by extension in the bigenic Tau.4RxGSK3*β* mice, was our most recent finding of the strict axonal location of Tau.4R, as opposed to the somatodendritic delocalization of Tau.P301L in transgenic mouse brain [92, 109].

### 7.2. Tau.P301L Mice

 In contrast to Tau.4R mice they exhibit less extensive phosphorylation of mutant protein Tau at younger age, but nevertheless develop into a representative model of tauopathy with neurofibrillary tangles and neuropil threads at older ages. These mice express the well-known human mutant Tau.P301L, the first to be associated with FTDP-17 [22, 23]. Human Tau.P301L is homozygously expressed under control of the same mouse Thy-1 gene promoter and in the same FvB/N genetic background as the Tau.4R mice [89].

**Figure 1:**
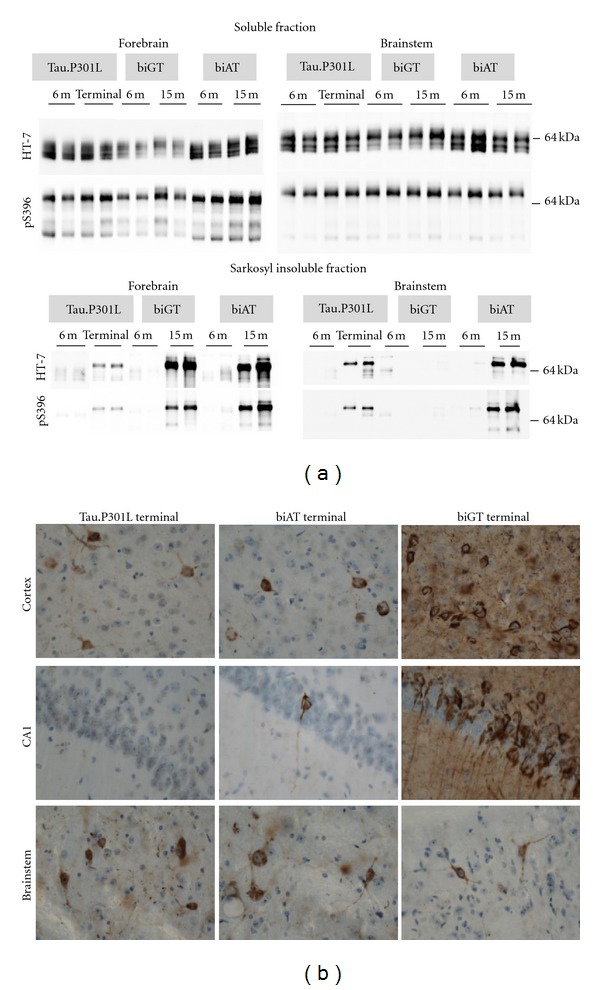
Selected biochemical and pathological characteristics of transgenic mouse models. (a) Soluble and sarkosyl insoluble fractions of Tau (SInT) were isolated as described [10] and proteins separated by SDS-PAGE on 10% tris-glycine gels. After transfer to nitrocellulose membranes, proteins were immunoblotted with either anti-pS396 (Invitrogen, Carlsbad, CA) or HT7 (Innogenetics, Gent, Belgium). Phosphorylated human Tau (apparent Mr about 64 kDa) is evident in soluble fractions from forebrain and brainstem of all three genotypes. SInT is evident in forebrain and brainstem of terminal TPLH and old biAT mice (age 15 months) but not in the brainstem of old biGT mice (15 months). (b) Immunohistochemistry with AT100 on free-floating sagittal sections of terminal mice shows tangles and neuropil threads in cortex and brainstem of all genotypes, but significantly less in the brainstem of biGT mice. Tauopathy is minimal or absent in pyramidal neurons of the hippocampus of terminal Tau.P301L transgenic mice.

Phosphorylation of Tau.P301L in mouse brain is initially low even up to the age of 7 months at the epitopes defined by Mabs AT8 and AT180, although phosphorylation is detectable with probes AD2 and AT270 [89]. The disease-associated epitope AT100 was even completely absent at young age. In older Tau.P301L mice, phosphorylation of Tau increases leading to reduced affinity of the MT-binding and consequent delocation to soma and dendrites. The local aggregation into tangles and neuropil threads in terminal mice is demonstrated immunohistochemically with Mab AT100 and others, for example, MC1 and PHF1, and biochemically by hyperphosphorylated Tau in the sarkosyl-insoluble fraction (Figure 1) [89]. The development of the tauopathy becomes associated with motor problems, illustrated by clasping and rotarod performance even as early as age 7.5 months, because the hindbrain and particularly specified nuclei in the brainstem, for example, Koelliker-Fuse and raphe, are hit by the tauopathy.

Most recently, we discovered the associated reduction of involuntary control of breathing, resulting in upper-airway defects that cause excessive workload of the diaphragm, leading to impaired ultrasonic vocalization, exhaustion, and asphyxia, most likely associated with dysphagia in old mice. These defects then explain the clinical moribund phenotype of terminal Tau.P301L mice, with dramatic reductions in bodyweight: males drop below 20–18 grams, females below 16 grams. Premature death occurs mainly in the time-window of 8–10 months with the mean around age 9.4 months, not dependent on gender, and with practically no survivors beyond age 12 months. The terminal stage evolves aggressively and takes less than 2-3 weeks after the first signs of motor impairment, whereby the mice rapidly lose body-weight, display a dystonic posture with progressive paralysis of all limbs and associated breathing problems leading to asphyxia, the presumed cause of premature death [11, 89, 110, 111].

Unexpectedly, the cognition of young Tau.P301L mice was markedly better than that of wild-type mice, with ameliorated long-term potentiation in the dentate gyrus and improved cognitive performance in object recognition tests [112]. This was anatomically substantiated by higher levels of mature spines in hippocampus and cortex compared to wild-type mice [109]. Although the spine maturation ratio remained high in hippocampus of adult Tau.P301L mice, the spines regress in length paralleling the impaired cognition, the increased phosphorylation and relocation of protein Tau.P301L from axons to soma and neuritic processes [109, 112].

Tau.P301L mice do not suffer any amyloid pathology and do not stand as a model for AD pathology, but these monogenic mice are an excellent model to study the impact of hyperphosphorylation and aggregation of protein Tau in different Tau pathologies. The progressive impairment that we documented in the Tau.P301L mice, starting from defective learning and memory in adults to problems at later age with breathing and vocalization, swallowing, movement, and motor defects, Tau phosphorylation, and aggregation into tangles, all correspond with features known from human tauopathies and neurodegenerative diseases. Needless to state that the effect of drugs or disease modifying treatments that target the pathological Tau, or its downstream partners or sequellae, can be evaluated in the monogenic Tau.P301L mice [19, 113].

### 7.3. biAT and biGT Mice

To define the relations and synergism between amyloid and Tau pathology in AD, and of the role of the GSK3 kinases, double transgenic mice were developed by combining homozygous Tau.P301L mice with either heterozygous human GSK3*β*.[S9A] mice or APP.V717I mice [77, 78, 114, 115], resulting, respectively, in GSK3*β*xTau.P301L (biGT) and APP.V717IxTau.P301L (biAT) bigenic mice. In both strains, the Tau pathology is aggravated with ageing by progressively increasing phosphorylation of Tau.P301L at typical pathological and conformational epitopes, resulting in the formation of highly fibrillary tangles and threads in cortex and hippocampus [77]. Conversely, in the brainstem of biGT mice the Tau pathology was significantly reduced relative to that in the parental Tau.P301L mice, which correlates with the markedly prolonged survival of the biGT mice compared to Tau.P301L mice: IC_50_ of 13 months versus 9.3 months, respectively.

Originally, we observed and described prolonged survival of the biAT mice, which was explained, by the increased activation of both GSK3 isozymes, that is, increased tyrosine phosphorylation, apparently driven by the high amyloid burden, as it was observed in the parental monogenic APP.V717I mice [77]. The subsequent in-depth further characterization of the biAT mice revealed a more complex pattern of mortality, which will be reported in detail elsewhere [116]. In brief, young biAT mice are prone to spontaneous or evoked epileptic seizures that cause a significant premature death in the tile-window of 1–6 months. Mortality is thereby evident at younger age and more frequent than in the parental APP.V717I mice [117].

Thereafter, mortality subsides, with a sizable fraction of biAT mice, particularly females surviving longer than the parental Tau.P301L mice. Of note, the female survivors develop, more severely than males, the typical combined AD-related pathology in relevant brain regions, that is, hippocampus, neocortex, entorhinal cortex, piriform cortex, and amygdala [77, 115]. In the forebrain of surviving biAT mice, the amyloid pathology with intracellular vesicular amyloid, extracellular diffuse and senile plaques, and vascular amyloid sets in at age from 10 to 12 months, preceding the Tau pathology. Of note, intracellular vesicular amyloid accumulation is already evident at young age (4–6 months) and is accompanied by increased phosphorylation of endogenous mouse Tau and of human Tau.P301L at the AT8 site. The timing remarkably coincides with the activation of the GSK3 kinases by intramolecular tyrosine phosphorylation and with the hippocampus-dependent cognitive deficits of APP.V717I and biAT mice. Cognition is already impaired at young adult age (age 4–6 months), well before the onset of amyloid deposition or Tau pathology, sensu stricto. Young adult biAT mice are severely impaired even in less demanding tests, that is, novel object recognition, passive inhibitory avoidance, and conditioned taste aversion [77].

Similar to humans suffering progressive senile types of dementia, ageing severely impacts the clinical phenotype of the bigenic mice, particularly of the biAT mice with progressive reduction in body weight, loss of ambulation, and reduced fur condition following the more early cognitive and behavioral impairments. The comparative evolution in both bigenic strains with ageing point to comparative underlying mechanisms, adding support to the hypothesis that amyloid triggers the tauopathy by increasing GSK3 activity [77, 118, 119].

## 8. Hypothesis: Tau, Phospho-Tau, Tau-P*, Oligomers, Fibrils, and Tangles

*“…a stage, and all the…merely players” W. Shakespeare 1598.*

The molecular characterization of protein species intermediate between the monomers prepared by biochemists and the large fibrils observed postmortem by the pathologists is a hot topic in current neuroscience research and for many proteins that are proven or surmised to be implicated in neurodegenerative disorder [15, 120–123]. Probably, amyloid is the most advanced in this respect, although no single intermediate A*β*-oligomer is yet accepted as the only or even major toxic species. Relying on thermodynamics and mass-action laws, one can even predict that a single species will never be identified as the proven cause of all the distress in AD brain over a period of 20 years, or more.

Pathological and experimental evidence increasingly questions the direct link between intraneuronal accumulation of Tau-aggregates and the neuronal degeneration observed in the later phases of dementia.

Many neurons that accumulate NFT in aged human brain and in transgenic mouse brain are not marked by characteristic morphological signs of cellular death. Conversely, many neurons that do bear such markers do not appear to have a significant load of high-order Tau filaments [44, 124].

**Figure 2:**
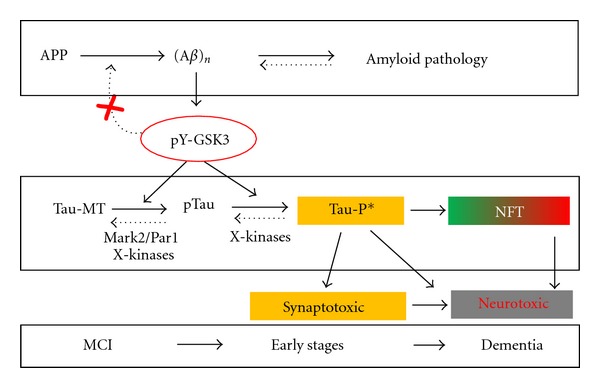
Schematic view of relations between amyloid and Tau in Alzheimer's disease. The interpretation hinges on the concept that the production of the Tau-P* intermediates are the central molecular species in the overall process. The incorporation of GSK3 as a major coupling link between the two pathologies is underscored essentially by *in vivo* observations in the mono- and bigenic mouse models discussed in the text. Protein Tau is a very soluble, naturally unfolded protein that in physiological conditions is located mainly in axons and attached to microtubules, denoted as Tau-MT. Activation by amyloid of both GSK3 kinases, together with other kinases including Mar2/Par1, gradually transforms Tau-MT into a pool of soluble, phosphorylated Tau (pTau) in the cytoplasm. Because of their delocalization, the pTau species undergo further posttranslational modifications, mainly including additional phosphorylation but eventually also nitrosylation, acetylation, truncation, all causing the transition into the conformational protein Tau species that we previously denoted as Tau-P* [11]. This as yet molecularly undefined intermediate likely represents soluble low-order aggregates that cause the early synaptic defects and cognitive problems typical for all tauopathies, with brain-region specificity as discussed in the text. The escape of Tau-P* from normal elimination via the proteasome and/or by autophagy leads to its accumulation, which by mass-action results in aggregation into NFT. We primarily consider NFT not to be detrimental for neurons, and initially they can even constitute a relative safety measure, as they reduce the free levels of Tau-P* and thereby its negative actions. Conversely, in the long term, the progressive accumulation of Tau-P* into more NFT deposition in neuropil and soma must invoke negative effects and eventually result in axonal and dendritic defects that culminate in neurodegeneration. This process is schematically reflected by the green-to-red color gradient of the background of the NFT box in the scheme.

Therefore, both tangle formation and neuron loss should be considered as dissociated processes, at least in time, but likely also in actual underlying mechanisms (Figure 2). Direct assessment of the relation of tangles and neuronal function by electrophysiology of individual neurons ex vivo, concomitant with structural analysis of dendritic branching and spines, did not correlate with NFT bearing [125]. Similarly, the findings in mammalian models was also observed in invertebrate Tau models [126, 127]. The justified conclusion must be that other, less aggregated or even soluble forms of protein Tau are responsible for neuronal dysfunction and by extension for failing synaptic plasticity and cognition. More and more, NFTs are regarded to function as the intraneuronal sink for excess phosphorylated protein Tau that was released from the axonal microtubules and after delocalization and extra phosphorylation is unable to regain its normal physiological position and function. By forming large fibrillar aggregates, neurons are protected from toxic effects of soluble, smaller aggregates [128, 129], a mechanism that was even proposed as a potential therapeutic approach [130]. Obviously, the cytoplasmic sink is limited in capacity and this protective measure cannot but be limited in time, eventually resulting in the damage and death of the neuron, exemplified by the presence of ghost tangles in the later stages of tauopathy [16].

Tau species responsible for cognitive dysfunction were tentatively identified as multimers of apparent Mr 170 kDa, present in FTD and AD brain, and correlating with memory index and motor deficits in tauopathy models [91]. Furthermore, Tau oligomers prepared with A*β* oligomers as initial seed invoked memory impairment and synaptic and mitochondrial dysfunction in wild-type mice [131]. Invertebrate transgenic models, for example, drosophila that express wild-type or mutant Tau isoforms also concluded to soluble cytosolic Tau species as accountable for toxicity [48, 132].

Conversely, our own experiments with AAV-based vectors in wild-type mice revealed early damage mainly to dendrites by human wild-type and mutant Tau alike, accompanied by extensive phosphorylation and smaller Tau-aggregates of Tau, but without formation of larger fibrils [47, 133]. The situation led us to postulate that a phosphorylated intermediate, most likely a dimer or small aggregate that we termed Tau-P*, is responsible for cognitive dysfunction by synaptic toxicity [19]. Our hypothesis hinges on the concept that the production of Tau-P* molecules constitutes for the neuron the tipping-point of whether or not to form the protective large aggregates. If for some reason, this is not possible, the accumulation of Tau-P* molecules will damage the neuron and impair synaptic function and plasticity, causing cognitive dysfunction (Figure 2).

This concept incorporates different signaling mechanism hinging on GSK3, and without abandoning the amyloid hypothesis. In the light of *in vivo* observations in transgenic mice, discussed in the foregoing sections, we maintain the GSK3 kinases center-stage as important link between the two pathologies in AD, reconciling the amyloid and Tau doctrines [77, 92, 119]. Activation of both GSK3 kinases by aberrant A*β* production and/or APP processing [134] is not the primary cause of the disease, but a major link to turn on the phosphorylation-cascade of protein Tau and the formation of Tau-P* intermediates. We realize that one kinase cannot suit all the known phosphorylation sites on protein Tau, while also the transformation of Tau-P* to NFT must depend on unknown factors (Figure 2). Moreover, the relation to the early and late cognitive impairment must depend on a host of as yet unknown molecular factors, termed X-factors before [19, 113] that must be held liable for the clinical symptoms in AD. Different factors in different individuals are expected to help explain the variable onset and evolution, as well as the variable cognitive and behavioral symptoms despite similar, although also variable brain pathology.
